# Bias-controlled multi-functional transport properties of InSe/BP van der Waals heterostructures

**DOI:** 10.1038/s41598-021-87442-1

**Published:** 2021-04-12

**Authors:** Sang-Hoo Cho, Hanbyeol Jang, Heungsoon Im, Donghyeon Lee, Je-Ho Lee, Kenji Watanabe, Takashi Taniguchi, Maeng-Je Seong, Byoung Hun Lee, Kayoung Lee

**Affiliations:** 1 School of Materials Science and Engineering, Gwangju Institute of Science and Technology (GIST), 123 Cheomdangwagi-ro, Buk-gu, Gwangju, 61005 Republic of Korea; 2 Department of Physics, Chung-Ang University, Seoul, 06974 Republic of Korea; 3 National Institute for Materials Science, 1-1 Namiki, Tsukuba, Ibaraki 305-0044 Japan; 4 Center for Semiconductor Technology Convergence (CSTC), Electrical Engineering, Pohang University of Science and Technology (POSTECH), 77 Cheongam-ro, Nam-gu, Pohang, Gyeongbuk 37673 Republic of Korea; 5 School of Electrical Engineering, Korea Advanced Institute of Science and Technology (KAIST), 291 Daehak-ro, Yuseong-gu, Daejeon, 34141 Republic of Korea

**Keywords:** Electrical and electronic engineering, Nanoscale devices

## Abstract

Van der Waals (vdW) heterostructures, consisting of a variety of low-dimensional materials, have great potential use in the design of a wide range of functional devices thanks to their atomically thin body and strong electrostatic tunability. Here, we demonstrate multi-functional indium selenide (InSe)/black phosphorous (BP) heterostructures encapsulated by hexagonal boron nitride. At a positive drain bias (*V*_D_), applied on the BP while the InSe is grounded, our heterostructures show an intermediate gate voltage (*V*_BG_) regime where the current hardly changes, working as a ternary transistor. By contrast, at a negative *V*_D_, the device shows strong negative differential transconductance characteristics; the peak current increases up to ~5 μA and the peak-to-valley current ratio reaches 1600 at *V*_D_ = −2 V. Four-terminal measurements were performed on each layer, allowing us to separate the contributions of contact resistances and channel resistance. Moreover, multiple devices with different device structures and contacts were investigated, providing insight into the operation principle and performance optimization. We systematically investigated the influence of contact resistances, heterojunction resistance, channel resistance, and the thickness of BP on the detailed operational characteristics at different *V*_D_ and *V*_BG_ regimes.

## Introduction

Heterogeneous electron systems consisting of van der Waals (vdW) materials have recently been receiving elevated attention, accompanied by the remarkable development of fabrication techniques^1–3^. The wide range of two-dimensional (2D) semiconductors with different band structure parameters presents the possibility of creating various band-edge alignments, depending on the desired device functionality. The absence of dangling bonds on the surfaces of 2D materials allows for high-quality heterointerfaces with minimal trap cites, regardless of lattice mismatch, and their atomically thin bodies render the band alignment properties and doping levels widely controllable by applying external electric fields^4,5^. vdW heterostructures, therefore, have revealed promise for advanced data processing electronics, including gate-tunable negative differential resistance, negative differential transconductance (NDT), and multi-value logic (MVL) operating devices^4–9^. For instance, Huang et al.^5^ demonstrated the multifunctionality of BP/MoS_2_ heterostructures, i.e. rectification, high on/off ratio, NDT, and ternary and binary logics. In-plane and interlayer tunneling electronics have also been realized based on various assemblies of low-dimensional semiconductors^6–12^.

NDT is a characteristic in which an increase in gate voltage results in a decrease in drain current; the so-called anti-ambipolar characteristic, one type of NDT, refers to a situation in which current is generated in the middle gate voltage region but is not generated at both positively and negatively large gate voltages^4,13^. Various heterostructures consisting of multiple different semiconductors have been shown to have NDT characteristics, including MoS_2_/single-walled carbon nanotubes^4^, MoS_2_/WSe_2_^6,13^, MoS_2_/MoTe_2_^14–16^, MoS_2_/BP^5^, ReS_2_/BP^17^, SnS_2_/WSe_2_^18^, MoS_2_/rubrene^19^, InSe/BP^20^, and heterostructures composed of *n*-type and *p*-type organic semiconductors^21,22^. A strong anti-ambipolar behavior can be employed for multi-way switching^13^. NDT devices also have potential use as oscillators, memories, and other low power logics^23,24^.

In this article, we present bias-controlled NDT and ternary *transistors* based on InSe/BP heterostructures encapsulated by hexagonal boron nitride (hBN). BP and InSe have mobilities notably superior than those of transition metal dichalcogenides^25,26^. They have relatively small effective masses, and the InSe conduction band and the BP valence band are close each other. The combination of InSe and BP could be, thus, promising for high-speed tunneling based devices. Several studies have demonstrated ternary *inverters* by using a NDT device and the part of the constituent semiconductor of the NDT device as a load resistor^5,6,14,22,27,28^. However, such approach is limited to inverting logics and cannot provide a versatile complementary metal–oxide–semiconductor (CMOS) circuit design strategy. Ternary *transistors*, where the drain current hardly changes within a specific gate voltage range (an intermediate logic state), are more essential to implement practical ternary-data-processing CMOS integrated circuits^29^.

InSe and BP yield different contact properties when contacted with Au/Ti contacts, and this asymmetry leads to asymmetric electrical characteristics depending on the polarity of drain bias (*V*_D_). While ternary states are developed at positive *V*_D_, NDT characteristics are observed at negative *V*_D_. Four-terminal measurements were performed on each layer, where, in principle, the contributions of contact resistances and channel resistance can be identified separately. We fabricated multiple devices with different contacts and device structures and systematically investigated the influence of contact resistances, heterojunction resistance, channel resistances, and the thickness of BP on the detailed operational characteristics.

## Results and discussion

**Figure 1:**
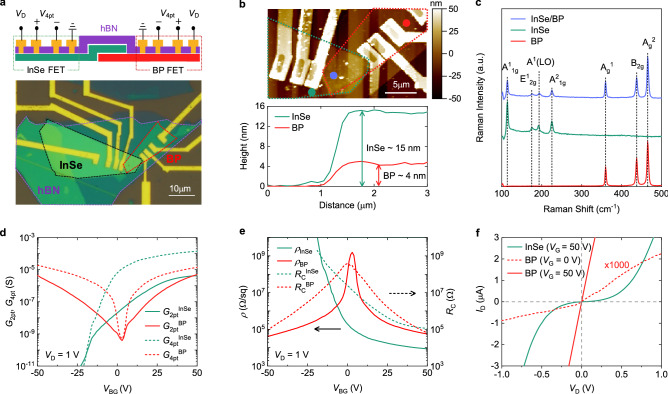
(**a**) Device schematic and optical micrograph of the InSe-BP-Ti device. The black, red, and purple dashed lines represent the InSe, BP, and hBN, respectively (lower micrograph). (**b**) Atomic force microscopic image of the device (upper) and the line profiles showing the thicknesses of the InSe and BP flakes (lower). (**c**) Raman spectra of the InSe/BP overlap region (blue), InSe region (green), and BP (red) region. (**d**) *G*_2pt_^InSe^, *G*_2pt_^BP^, *G*_4pt_^InSe^, and *G*_4pt_^BP^ measured via the four-terminal methodology as a function of *V*_BG_ at *V*_D_ = 1 V. e) *R*_C_^InSe^, *R*_C_^BP^, *ρ*_InSe_, and *ρ*_BP_ extracted from (**d**) data. (**f**) *I*_D_ vs *V*_D_ of the InSe at *V*_BG_ = 50 V and that of the BP at *V*_BG_ = 0 and 50 V, showing the Schottky behavior of the Au/Ti contacts on the InSe and the ohmic characteristic of the contacts on the BP.

Figure 1a describes the structure of our hBN/InSe/BP heterostructure device with Au/Ti electrodes (InSe-BP-Ti device), accompanied by its optical micrograph. The hBN/InSe/BP heterostructure was achieved using the conventional dry transfer method^2,3^. Individually exfoliated BP and InSe flakes were successively transferred on 300 nm-thick SiO_2_, followed by encapsulation with a pre-patterned hBN flake. InSe and BP are vulnerable to the air, but the hBN encapsulation allows improved stability and decent mobility^26,30^. Before the transfer, the hBN encapsulating layer was patterned using electron beam (e-beam) lithography and plasma etching to have eight openings. These allowed the InSe and BP to be partially exposed for metallization even after having the hBN cover on them. Four metal contacts on the InSe and BP were then defined by e-beam lithography, followed by Au(80 nm)/Ti(10 nm) deposition and lift-off. Figure 1b shows the atomic force microscopy (AFM) image, which reveals the thicknesses of the InSe and BP flakes as 15 nm and 4 nm, respectively. Figure 1c shows the three distinct Raman spectra measured on the InSe, BP, and InSe/BP overlap regions (excitation wavelength = 514 nm). The four peaks at 114 cm^−1^, 175 cm^−1^, 198 cm^−1^, and 225 cm^−1^ from InSe, and the three peaks at 364 cm^−1^, 440 cm^−1^, and 467 cm^−1^ from BP are consistent with those previously reported^31,32^, confirming the high quality of our flakes encapsulated by hBN. The Raman spectrum on the overlapped region shows the same peak positions originating from the InSe and BP. The Raman peak intensity is slightly weakened in the InSe/BP overlap region, compared to that in InSe/SiO_2_. This Raman quenching is attributed to the weak but finite van der Waals coupling between the InSe and BP^33,34^. Nearly consistent peak positions and the ratio of A^1^(LO) peak to E^1^_2g_ peak intensity suggest that the degree of strain and doping induced by having the heterostructure is negligible^30^.

Electrical characterization was performed on the BP and InSe individually via the two-terminal and four-terminal methodology, as illustrated in the schematic in Fig. 1a^26,35^. Figure 1d shows the two-terminal conductances (*G*_2pt_) of the InSe (*G*_2pt_^InSe^) and BP (*G*_2pt_^BP^), and the channel conductances (*G*_4pt_) of the InSe (*G*_4pt_^InSe^) and BP (*G*_4pt_^BP^) as a function of back-gate voltage (*V*_BG_), where *G*_2pt_ = *I*_D_/*V*_D_, *G*_4pt_ = (*I*_D_/*V*_4pt_)(*L*_4pt_/*L*_2pt_), *I*_D_ is the measured drain current, *V*_D_ is the drain bias applied on the InSe or BP, *V*_4pt_ is the voltage difference between the two inner contacts on the InSe or BP, and *L*_4pt_ (*L*_2pt_) is the distance between the two inner (outer) contacts. While the BP shows an ambipolar characteristic, the InSe shows an *n*-type semiconducting behavior, similar to those of prior reports^25,26^.

We note that the two-terminal field-effect mobilities of the InSe and BP are ~20 cm^2^ V^−1^ s^−1^ and ~65 cm^2^ V^−1^ s^−1^, respectively, and the four-terminal field-effect mobilities of the InSe and BP are ~370 cm^2^ V^−1^ s^−1^ and ~120 cm^2^ V^−1^ s^−1^, respectively (Figure S1). The two-terminal mobilities are obtained via ((1/*C*)(*L*_2pt_/*W*)(*dG*_2pt_/*dV*_BG_)) and the four-terminal mobilities are obtained via ((1/*C*)(*L*_2pt_/*W*)(*dG*_4pt_/*dV*_BG_)), where *C* is the back-gate capacitance and *W* is the channel width. The two-terminal mobilities are smaller than the four-terminal values due to contact resistances^36^, as is particularly notable for the InSe. Figure 1e shows the channel resistivities (*ρ*) of the InSe (*ρ*_InSe_) and BP (*ρ*_BP_), and the contact resistances (*R*_C_) for the InSe (*R*_C_^InSe^) and BP (*R*_C_^BP^), where *ρ* = (1/*G*_4pt_)(*W*/*L*_2pt_) and *R*_C_ = (1/*G*_2pt_ − 1/*G*_4pt_)/2^37^. *R*_C_^BP^ (*R*_C_^InSe^) occupies a large portion of the two-terminal resistance, 1/*G*_2pt_^BP^ (1/*G*_2pt_^InSe^). Figure 1f also provides *I*_D_ vs *V*_D_ output characteristics for the InSe and BP. The BP shows a linear dependence at high carrier density and even at charge neutrality (at *V*_BG_ = 50 V and 0 V), suggesting ohmic contacts, but the InSe shows a nonlinear characteristic, indicating Schottky contacts.

**Figure 2:**
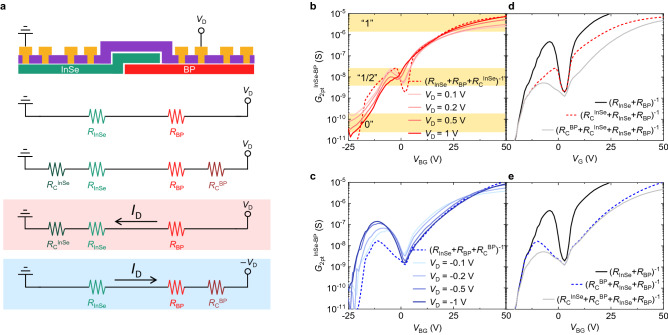
(**a**) Schematics describing the InSe-BP-Ti device (upper) and the four different equivalent circuits, consisting of *R*_InSe_, *R*_BP_, *R*_C_^InSe^, and *R*_C_^BP^. Depending on current direction, different contact resistance components are more effective. (**b**) *G*_2pt_^InSe−BP^ vs *V*_BG_ of the InSe-BP-Ti device measured at a forward *V*_D_ regime, which shows a distinct intermediate logic state with a small fluctuation. The dashed line represents (*R*_InSe_ + *R*_BP_ + *R*_C_^InSe^)^−1^, which is comparable to the measured *G*_2pt_^InSe−BP^. (**c**) *G*_2pt_^InSe−BP^ vs *V*_BG_ of the InSe-BP-Ti device measured at a backward *V*_D_ regime, which shows a strong NDT behavior with PVCR reaching ~ 10^2^ at *V*_D_ = −1 V. The dashed line represents (*R*_InSe_ + *R*_BP_ + *R*_C_^BP^)^−1^, which is comparable to the *G*_2pt_^InSe−BP^. d,e) (*R*_InSe_ + *R*_BP_)^−1^, (*R*_InSe_ + *R*_BP_ + *R*_C_^InSe^)^−1^, (*R*_InSe_ + *R*_BP_ + *R*_C_^BP^)^−1^, and (*R*_InSe_ + *R*_BP_ + *R*_C_^BP^ + *R*_C_^InSe^)^−1^ for comparison with (**b,c**) data, respectively.

**Figure 3:**
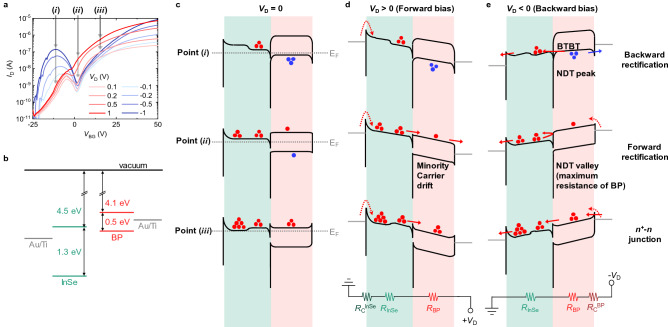
(**a**) *I*_D_ versus *V*_BG_ of the InSe-BP-Ti device at different *V*_D_ values. Characteristic points (i), (ii), and (iii) are marked. (**b**) Schematic showing the band parameters and alignment between the InSe, BP, and both Au/Ti contacts. Previously reported Schottky barrier heights of the Au/Ti contacts for the InSe and BP, rather than metal work functions, are marked^43,44^. (**c–e**) Band diagrams of the InSe-BP-Ti device at points (i), (ii), and (iii) and at (**c**) *V*_D_ = 0, (**d**) *V*_D_ > 0, and (**e**) *V*_D_ < 0. Red dots represent electrons, and blue dots represent holes. Corresponding equivalent circuits are illustrated in (**d,e**).

Figure 2a describes the measurement setup for the bias-controlled MVL and NDT properties of the InSe-BP-Ti device. While applying *V*_D_ to the BP and grounding the InSe, the two-terminal conductance between the source and drain (*G*_2pt_^InSe−BP^ = *I*_D_/*V*_D_) was measured in our device. Figure 2b,c show the measured *G*_2pt_^InSe−BP^ as a function of *V*_BG_ at positive and negative *V*_D_, respectively (Fig. 3a also provides the corresponding *I*_D_ as a function of *V*_BG_). In the positive *V*_D_ regime, we note the particular region where the *G*_2pt_^InSe−BP^ hardly changes, creating an intermediate state “1/2” (at −6 V < *V*_BG_ < 0), accompanied by a state “0” (at *V*_BG_ < −20 V) and a state “1” (at *V*_BG_ > 25 V). Several studies have demonstrated ternary *inverters* based on NDT^5,6,14,22,27,28^, where the potential inside of the constituent semiconductor barely changes within the negative transconductance region. However, this methodology is limited to the inverting logic and indeed not suitable for versatile CMOS circuitry design. Ternary *transistors* are indispensable to design versatile ternary-data-processing CMOS integrated circuits. In the negative *V*_D_ regime, a strong NDT behavior is observed, achieving a peak conductance (*G*_peak_) of 140 nS and a peak-to-valley current ratio (PVCR) of ~ 10^2^ at *V*_D_ = −1 V. Figure S2 shows transfer characteristics of our another InSe-BP-Ti device, which are qualitatively similar to those in Fig. 2b,c.

In the InSe-BP-Ti device, the total two-terminal resistance from source to drain can be approximately modeled as serially connected resistors (Fig. 2a), which can include *R*_C_^InSe^, *R*_C_^BP^, the channel resistances of the InSe (*R*_InSe_), and of the BP (*R*_BP_). Each of these components is acquired via the four-terminal measurements at *V*_D_ = 1 V, as discussed in Fig. 1e. *R*_InSe_ (*R*_BP_) corresponds to *ρ*_InSe_ (*ρ*_BP_) multiplied by the appropriate dimension of the InSe (BP) region of the InSe-BP-Ti device; Figure S3 provides schematics and a table to define the multiple parameters we introduced more specifically. We assume that most of the current flows along the InSe rather than the BP in the InSe/BP overlap region. The InSe region that sits above the BP is not effectively gate-controlled due to the thick BP underneath, and thus, the InSe resistivity is assumed to be constant as *ρ*_InSe_ at *V*_BG_ = 0 V, which is smaller than *ρ*_BP_ regardless of the applied *V*_BG_ (Fig. 1e). We also remark that the resistance at the heterojunction between the InSe and BP is not taken into account, which is further discussed below.

As described in the lower schematics of Fig. 2a, four different equivalent circuits are compared with our experimental data: (1) *R*_InSe_ + *R*_BP_, (2) *R*_C_^InSe^ + *R*_InSe_ + *R*_BP_ + *R*_C_^BP^, (3) *R*_C_^InSe^ + *R*_InSe_ + *R*_BP_, and (4) *R*_InSe_ + *R*_BP_ + *R*_C_^BP^. We present conductance rather than drain current in Fig. 2b,c for the comparison of the measurement to these series circuit models; there exist finite errors between them, which are discussed below. In the case of (*R*_InSe_ + *R*_BP_)^−1^ without any *R*_C_ (black solid in Fig. 2d,e), a superior NDT peak arises at *V*_BG_ ~ −4 V, which simply corresponds to when *R*_BP_ + *R*_InSe_ becomes a minimum in the *p*-side of the BP (see Fig. 1d). When *V*_BG_ < −4 V, the (*R*_InSe_ + *R*_BP_)^−1^ value is strongly governed by *R*_InSe_, which is in an insulating state. As the *V*_BG_ increases, *R*_InSe_ decreases and correspondingly (*R*_InSe_ + *R*_BP_)^−1^ increases, reaching the NDT peak at *V*_BG_ ~ −4 V. (*R*_InSe_ + *R*_BP_)^−1^ then rapidly decreases and shows a valley as the BP reaches its charge neutrality, which is followed by an increase of (*R*_InSe_ + *R*_BP_)^−1^ as the *n*-side of the BP turns on with increasing *V*_BG_. The overall characteristic behavior of (*R*_BP_ + *R*_InSe_)^−1^ is analogous to that of our experimentally measured *G*_2pt_^InSe−BP^ at negative *V*_D_, but the *G*_peak_ of (*R*_BP_ + *R*_InSe_)^−1^ is much higher than the experimental values. By contrast, the calculated (*R*_C_^InSe^ + *R*_InSe_ + *R*_BP_ + *R*_C_^BP^)^−1^ value, including the *R*_C_ components, yields a largely reduced *G*_peak_, which is even lower than the experimental values at both negative and positive *V*_D_ regimes.

Depending on the polarity of *V*_D_, the *R*_C_ on the other side (*R*_C_^InSe^ or *R*_C_^BP^) can be more effective due to the asymmetric nature of Schottky barriers^38,39^. In particular, the channel of our InSe-BP-Ti device consists of two different materials, InSe and BP, which leads to strong asymmetric characteristics, depending on the polarity of *V*_D_. Interestingly, the (*R*_C_^InSe^ + *R*_InSe_ + *R*_BP_)^−1^ is similar to the measured *G*_2pt_^InSe−BP^ at positive *V*_D_, while the (*R*_InSe_ + *R*_BP_ + *R*_C_^BP^)^−1^ is similar to the measured *G*_2pt_^InSe−BP^ at negative *V*_D_. This is because when *V*_D_ > 0, electrons generally see a barrier at the junction between the Au/Ti contact and the InSe^40^. By contrast, when *V*_D_ < 0, carriers generally see a barrier at the junction between the contact and the BP. The band diagrams further describe which side of the contact resistances, *R*_C_^InSe^ or *R*_C_^BP^, is more effective, depending on the polarity of *V*_D_ (Fig. 3b–e).

Figure 3b represents the band parameters we used to build the band diagrams^41–43^. The Schottky barrier for electrons at the junction between the 4-nm thick BP and Au/Ti contact was presumed to be ~0.2 eV^43^, and the Schottky barrier at the junction between the InSe and Au/Ti contact was roughly presumed to be ~0.3 eV^44^, as previously reported. Figure 3c–e describe the band diagrams at *V*_D_ = 0, *V*_D_ > 0, and *V*_D_ < 0 and at points (i), (ii), and (iii), marked in Fig. 3a. At *V*_D_ > 0 (Figs. 2b, 3d), the electrons entering the InSe/BP channel experience a barrier at the junction between the source and the InSe, resulting in *R*_C_^InSe^. The slightly smaller *G*_2pt_^InSe−BP^ compared to the (*R*_C_^InSe^ + *R*_InSe_ + *R*_BP_)^−1^ at point (i) is presumably due to the underestimated *R*_C_^InSe^ value in the subthreshold region of the InSe. As the *V*_BG_ decreases, the *R*_C_^InSe^ dramatically increases (Fig. 1e)^26^, exceeding the reasonable range measurable via the conventional four-terminal methodology. The slightly higher *G*_2pt_^InSe−BP^, compared to the (*R*_C_^InSe^ + *R*_InSe_ + *R*_BP_)^−1^ at point (ii), is due to the minority carriers drifting in the BP injected from the InSe, a typical characteristic of a *p*-*n* diode at a forward bias. *G*_2pt_^InSe−BP^ agrees well with (*R*_C_^InSe^ + *R*_InSe_ + *R*_BP_)^−1^ at point (iii), where the *n*^+^-*n* junction is made. At *V*_D_ < 0 (Figs. 2c, 3e), electron carriers generally see a barrier at the junction between the drain and the BP, experiencing *R*_C_^BP^. Distinctively, at point (i), electrons can be injected from the BP valence band, full of electrons, into the InSe via band-to-band tunneling (BTBT)^11^. This leads to the reduced impact of *R*_C_^BP^ and the corresponding enhanced NDT peak compared to (*R*_InSe_ + *R*_BP_ + *R*_C_^BP^)^−1^ (Fig. 2c). At points (ii) and (iii), the measured *G*_2pt_^InSe−BP^ agrees well with (*R*_InSe_ + *R*_BP_ + *R*_C_^BP^)^−1^. The band diagrams also explain the backward rectification at point (i) and the forward rectification at point (ii). The rectification ratio (*I*_D_ at positive *V*_D_ divided by *I*_D_ at negative *V*_D_) values of our multiple InSe-BP-Ti devices are provided in Figure S4, where the backward rectification ratio reaches up to 10^5^.

**Figure 4:**
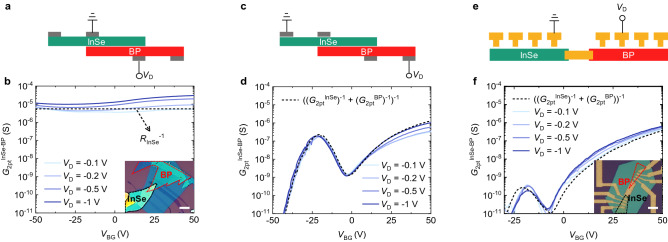
(**a**) Schematic describing the InSe-BP-FLG device and the measurement setup using the inner two electrodes. (**b**) *G*_2pt_^InSe−BP^ of the InSe-BP-FLG device measured as a function of *V*_BG_ at different *V*_D_ values, corresponding to the conductance of the InSe/BP overlap region. The inset shows an optical micrograph of the InSe-BP-FLG device (scale bar = 10 μm). (**c**) Schematic showing the measurement setup using the outer two electrodes of the InSe-BP-FLG device. (**d**) *G*_2pt_^InSe−BP^ of the InSe-BP-FLG device as a function of *V*_BG_ at different *V*_D_ values, corresponding to the conductance over the whole channel region, including the non-overlapped InSe and BP regions. (**e**) Schematic describing the InSe-Ti-BP-Ti device and corresponding measurement setup. (**f**) *G*_2pt_^InSe−BP^ of the InSe-Ti-BP-Ti device as a function of *V*_BG_ at different *V*_D_ values. The inset shows an optical micrograph of the InSe-Ti-BP-Ti device (scale bar = 10 μm).

We investigated multiple InSe-BP devices with different contacts and structures: (1) an InSe/BP heterostructure device with few-layer graphene (FLG) contacts (InSe-BP-FLG device, Fig. 4a–d), and (2) another device where InSe and BP are serially connected via Au/Ti contacts (InSe-Ti-BP-Ti device, Fig. 4e,f). As described in Fig. 4a, there are two FLG contacts on the InSe and two others under the BP in the InSe-BP-FLG device. Figure 4b shows the *G*_2pt_^InSe−BP^ measured using the inner two contacts, where the InSe and BP are vertically overlapped over the whole measured region. It is notable that the *G*_2pt_^InSe−BP^ barely depends on *V*_BG_, without NDT behavior. The InSe-BP-FLG device is also measured using the outer two contacts as source and drain (Fig. 4c). In contrast to the Fig. 4a setup, the individual non-overlapped InSe and BP regions are included in the Fig. 4c setup. Figure 4d shows the correspondingly measured *G*_2pt_^InSe−BP^ as a function of *V*_BG_. Note the NDT behavior, similar to that observed in the InSe-BP-Ti device at a negative *V*_D_ regime. This contrasts remarkably with the *G*_2pt_^InSe−BP^ in Fig. 4b, measured only in the InSe/BP overlap region. This clear distinction shows that the non-overlapped regions play a major role in inducing the NDT behavior. The measured *G*_2pt_^InSe−BP^ in Fig. 4d agrees well with ((*G*_2pt_^InSe^)^−1^ + (*G*_2pt_^BP^)^−1^)^−1^ (dashed), without considering the InSe/BP vdW junction resistance. The thicknesses of the BP and InSe layers used for the InSe-BP-FLG device are 4 nm and 15 nm (Figure S5), respectively, similar to those of the InSe-BP-Ti device. The *G*_2pt_^InSe−BP^ in Fig. 4b indeed agrees well with the *R*_InSe_^−1^ (dashed) estimated using the *ρ*_InSe_ at *V*_BG_ = 0 V (data in Fig. 1e) and the dimension of the InSe region between the two inner contacts, without taking into account any of the BP region and the InSe/BP junction resistance. This suggests that the current flows mostly along the InSe, and the heterojunction resistance and FLG contact resistances are negligible compared to the InSe channel resistance. We thus neglect the InSe/BP junction resistance when considering the equivalent circuits of the InSe-BP-Ti device in Fig. 2. The low InSe/BP junction resistance even when the BP is *p*-type (at *V*_BG_ < 0) and *V*_D_ < 0 is due to BTBT between the InSe and BP, as described in the band diagram in Fig. 3e. The output characteristics of the individual InSe and BP regions with FLG contacts also reveal ohmic characteristics (Figure S6), which contrasts with the Schottky behavior of Au/Ti contacts on the InSe. This leads to the transconductance behavior being nearly the same regardless of the polarity of *V*_D_ (Figure S7), distinct from the InSe-BP-Ti device.

The schematic in Fig. 4e describes the InSe-Ti-BP-Ti device where InSe and BP are serially connected via Au/Ti contacts and the associated measurement setup. Figure 4f shows the *G*_2pt_^InSe−BP^ measured from the InSe-Ti-BP-Ti device as a function of *V*_BG_ at different *V*_D_, ranging from -0.1 to -1 V. The NDT characteristics are also observed in this device, similar to the InSe-BP-Ti device and InSe-BP-FLG device. This further reveals that the NDT behavior originates from the individual InSe and BP parts rather than the vertically overlapped InSe/BP region. The measured *G*_2pt_^InSe−BP^ agrees well with ((*G*_2pt_^InSe^)^−1^ + (*G*_2pt_^BP^)^−1^)^−1^ (dashed line in Fig. 4f). However, we note the much lower *G*_peak_ (~0.1 nS), compared to those of the InSe-BP-Ti device and the InSe-BP-FLG device (>0.1 μS). This is due to the additional Au/Ti contact resistances that exist between the InSe and BP, which are much larger than the InSe/BP vdW junction resistance. The thicknesses of the BP and InSe flakes in the InSe-Ti-BP-Ti device were 3 nm and 9 nm, respectively (Figure S8).

**Figure 5:**
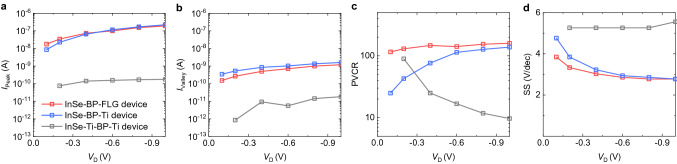
(**a**) *I*_Peak_, (**b**) *I*_Valley_, (**c**) PVCR, and (**d**) SS of the InSe-Ti-BP-Ti (grey), InSe-BP-Ti (blue), and InSe-BP-FLG (red) devices as a function of *V*_D_.

Figure 5a,b show the peak current (*I*_Peak_) and valley current (*I*_Valley_) of the InSe-BP-Ti, InSe-BP-FLG, and InSe-Ti-BP-Ti devices as a function of *V*_D_ from −0.1 to −1 V. Both *I*_Peak_ and *I*_Valley_ values of the InSe-Ti-BP-Ti device are far lower than those of the InSe-BP-Ti and InSe-BP-FLG devices. As mentioned above, this is attributed to the additional *R*_C_^InSe^ and *R*_C_^BP^ from the Au/Ti contacts between the InSe and BP. Figure 5c,d show the extracted PVCR and subthreshold swing (SS) values of the devices, respectively. While the PVCR of the InSe-BP-Ti device increases as the *V*_D_ increases, the PVCR of the InSe-BP-FLG device weakly depends on *V*_D_. In the InSe-BP-Ti device, as *V*_D_ increases, *R*_C_^BP^ decreases; thus, the PVCR increases and the SS decreases. By contrast, the low FLG contact resistance in the InSe-BP-FLG device leads to the PVCR and SS being less dependent on *V*_D_.

The transfer characteristics of the InSe-Ti-BP-Ti, InSe-BP-Ti, and InSe-BP-FLG devices can be compared to those calculated as (*R*_InSe_ + *R*_C_^InSe^ + *R*_BP_ + *R*_C_^BP^)^−1^, (*R*_InSe_ + *R*_BP_ + *R*_C_^BP^)^−1^, and (*R*_InSe_ + *R*_BP_)^−1^, respectively (Fig. 2a). The calculated conductances at their NDT valley points are similar (Fig. 2e), regardless of the inclusion of *R*_C_^InSe^ or *R*_C_^BP^. This is because the valley point characteristic is dominated by the large *R*_BP_ at the charge neutrality of BP. On the other hand, the conductance at the NDT peak strongly depends on whether *R*_C_^InSe^ or *R*_C_^BP^ is included. The additional *R*_C_^InSe^ and *R*_C_^BP^ existing in the InSe-Ti-BP-Ti device result in much lower PVCR and worse SS values due to reduced gate control. By contrast, the negligible vdW junction resistance renders better PVCR and SS values for the InSe/BP vdW heterostructure devices.

In many cases, ohmic contacts are more favorable than Schottky contacts. Our InSe-BP-FLG device also shows *I*_Peak_ and PVCR values higher than those of InSe-BP-Ti device (Fig. 5). The ohmic symmetric contacts in InSe-BP-FLG device lead to the nearly the same NDT characteristics for both positive and negative *V*_D_ regimes (Figure S7). On the other hand, the asymmetric Au/Ti contacts to the InSe and BP in InSe-BP-Ti device can provide dissimilar operational properties depending on the polarity of *V*_D_, multi-valued transistor behavior at *V*_D_ > 0 and NDT at *V*_D_ < 0. Particularly at *V*_D_ > 0, the high Schottky barrier (contact resistance) at the junction between Au/Ti contact and InSe becomes highly effective (point (i) in Fig. 3). This suppresses the current in that regime, leading to the intermediate state for the multi-valued transistor instead of a NDT peak (Figs. 2, 3, and Figure S2). Our study as a function of multiple device parameters, including contact type and heterojunction type, can provides an idea for fine manipulation of electronic device characteristics.

**Figure 6:**
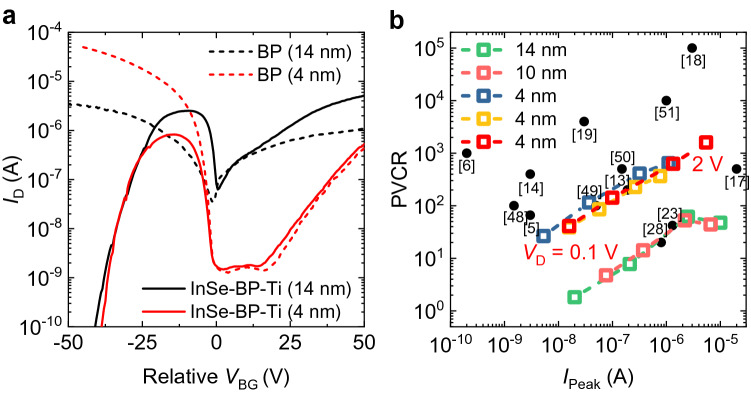
(**a**) *I*_D_ versus *V*_BG_ transfer characteristics of the InSe-BP-Ti devices (solid) and the BP (dashed) with different BP thicknesses, which are presented in parentheses. The *V*_BG_ is shifted relative to the minimum current point of the BP for each device. (**b**) PVCR versus *I*_Peak_ characteristics of our multiple InSe-BP-Ti devices with different BP thicknesses, accompanied by previously reported PVCR values of other 2D material–based NDT devices for comparison.

In addition, we investigated the impact of BP thickness (*t*_BP_) on the electrical characteristics of our InSe-BP-Ti devices. Figure 6a shows the *I*_D_ of two InSe-BP-Ti devices (solid) with different *t*_BP_, 4 nm and 14 nm, as a function of *V*_BG_, shifted with respect to the *V*_BG_ at the minimum *I*_D_ of the BP. The most salient feature here is that the device with thinner BP shows significantly reduced *I*_Valley_, resulting in a highly improved PVCR. The transfer curve of the BP side of each InSe-BP-Ti device is also provided for comparison (dashed). The on/off current ratio of the 4 nm-thick BP is much higher than that of the 14 nm-thick BP due to the increased band gap, as previously reported^45^. Depending on the BP thickness ranging from 14 to 4 nm, the band gap of BP varies from 0.3 to 0.5 eV^43^. The BP band gap change from 0.3 to 0.5 eV leads to a notable impact on the off current and corresponding PVCR of the NDT. On the other hand, the band gap change of InSe of similar thickness (4–14 nm) is 1.3–1.4 eV^46^, and it does not cause much change in the off current because the band gap within that range is already quite large, compared to *kT*; *k* is the Boltzmann constant and *T* is the temperature. Further thicker InSe might lead to improved *I*_Peak_ as the mobility increases for thicker InSe^47^. Multiple InSe-BP-Ti devices with different *t*_BP_, ranging from 4 to 14 nm, were examined. Figure 6b shows their PVCR values versus *I*_Peak_ at different *V*_D_, along with previously reported PVCR values of other 2D material–based NDT devices^5,6,13,14,17–19,23,28,48–51^. As noted above, the InSe-BP-Ti devices with thinner BP show higher PVCR values compared to the devices with thicker BP flakes thanks to the higher band gap and corresponding higher on/off ratio of the BP. *I*_Peak_ and PVCR further increase with increasing *V*_D_, as seen in Fig. 5, achieving *I*_Peak_ of ~5 μA and PVCR of ~ 1600 at *V*_D_ = −2 V (Figure S9). The performance also depends on the mobility and the initial doping level of BP. A few studies have reported PVCR values higher than ours, but these either applied much higher *V*_D_ (≥10 V) or had a wider *V*_BG_ width of the peak^18,19^.

## Conclusion

In summary, we demonstrated bias-controlled MVL and NDT properties based on InSe/BP heterostructures. Due to the asymmetric nature of Schottky barriers at the junction between Au/Ti contact and InSe or BP, asymmetric electrical characteristics were developed depending on the polarity of *V*_D_. At positive *V*_D_, the InSe-BP-Ti devices worked as ternary transistors, but at negative *V*_D_, the devices showed NDT characteristics. The contributions of contact resistances and channel resistance were identified separately via four-terminal measurements performed on each layer. Multiple devices with different contacts and device structures were investigated, and we systematically discussed the influence of contact resistances, heterojunction resistance, channel resistances, and the thickness of BP on the detailed operational characteristics of InSe/BP-based vdW heterostructures. These results provide insight into the operation principle and further performance optimization of general 2D material–based vdW heterostructures.

## Experimental

### Fabrication of the InSe-BP-Ti device

InSe and BP flakes were mechanically exfoliated on polydimethylsiloxane (PDMS) film using cleanroom tape, while hBN was exfoliated on 300 nm-thick SiO_2_/Si substrate. Once appropriate InSe and BP flakes with the desired thicknesses were identified, the PDMS films with the flakes were cut into 10 mm × 10 mm pieces and then attached to a glass slide for transfer to a designated location. The BP and InSe flakes on PDMS film were then successively transferred on 300 nm-thick SiO_2_, thermally grown on a highly doped Si substrate, resulting in an InSe/BP stack. To minimize the surface contamination of the device, the exfoliation and stacking processes were performed entirely in a glovebox, keeping the oxygen level below 5 ppm. The separately exfoliated hBN flake suitable for top encapsulation was patterned before the transfer using conventional e-beam lithography and reactive ion etching using SF_6_ gas to have eight openings. These allowed the InSe and BP to be partially exposed for metallization even after having the hBN cover on them. The patterned hBN flake was annealed at 400℃ for an hour in the Ar/H_2_ atmosphere to remove polymethyl methacrylate (PMMA) residue, which also allowed the hBN to be easily picked up using a polypropylene carbonate (PPC)/PDMS stamp. The patterned hBN was then transferred onto the InSe/BP stack by melting the PPC at ~120 ℃. Then the hBN/InSe/BP heterostructure was washed with acetone. Additional e-beam lithography was performed to define metal contacts for the InSe-BP-Ti device, followed by Au(80 nm)/Ti(10 nm) deposition via e-beam evaporation and lift-off. The device was annealed at 400℃ for an hour in the Ar/H_2_ atmosphere to remove the remaining polymer residues.

### Fabrication of the InSe-Ti-BP-Ti device

The fabrication process for the InSe-Ti-BP-Ti device was similar to that of the InSe-BP-Ti device. One distinction was that the BP and InSe flakes were ~5 μm apart from each other without InSe/BP vdW heterojunction.

### Fabrication of the InSe-BP-FLG device

FLG and hBN flakes were mechanically exfoliated on Si/SiO_2_ substrates. They were annealed at 400°C for an hour in the Ar/H_2_ atmosphere. Individually exfoliated BP and InSe flakes were successively transferred on the FLG exfoliated on SiO_2_, similar to the transfer process employed for the InSe-BP-Ti device. Finally, hBN and top FLG flakes were successively picked up using a PPC/PDMS stamp. Then the hBN/FLG stack was transferred to the prepared InSe/BP/FLG heterostructure on SiO_2_. Au/Ti contacts were deposited on a non-encapsulated region of the FLG, followed by annealing in the Ar/H_2_ atmosphere.

### Material and electrical characterization

The surface topography and material quality of the fabricated devices were examined via AFM (Park systems, XE-100) and Raman spectroscopy (Renishaw, 514 nm wavelength laser). The electrical measurements were performed using a semiconductor analyzer (Keithley, 4200A-SCS) at high vacuum (~10^–6^ bar).

## Supplementary Information

Supplementary Figures.
