# Supplementary material for: Bias-controlled multi-functional transport properties of InSe/BP van der Waals heterostructures
Source: Sci Rep. 2021 Apr 12;11:7843. doi: 10.1038/s41598-021-87442-1 (PMC8041794; doi:10.1038/s41598-021-87442-1)
Supplement: Supplementary file 1 — Supplementary Figures. [file 41598_2021_87442_MOESM1_ESM.pdf]

Supplementary material

Bias-controlled Multi-functional Transport Properties of InSe/BP van der Waals Heterostructures

Sang-Hoo Cho[1], Hanbyeol Jang[1], Heungsoon Im[1], Donghyeon Lee[1], Je-Ho Lee[2], Kenji Watanabe[3], Takashi Taniguchi[3], Maeng-Je Seong[2], Byoung Hun Lee[1,4], Kayoung Lee[1,5,*]

[1]School of Materials Science and Engineering, Gwangju Institute of Science & Technology (GIST), 123 Cheomdangwagi-ro, Buk-gu, Gwangju 61005, Republic of Korea

[2]Department of Physics, Chung-Ang University, Seoul 06974, Republic of Korea

[3]National Institute for Materials Science, 1-1 Namiki Tsukuba Ibaraki 305-0044, Japan

[4]Center for Semiconductor Technology Convergence (CSTC), Electrical Engineering, Pohang University of Science & Technology (POSTECH), 77 Cheongam-ro, Nam-gu, Pohang, Gyeongbuk 37673, Republic of Korea

[5]School of Electrical Engineering, Korea Advanced Institute of Science & Technology (KAIST), 291 Daehak-ro, Yuseong-gu, Daejeon 34141, Republic of Korea

*kayoung.lee@kaist.ac.kr

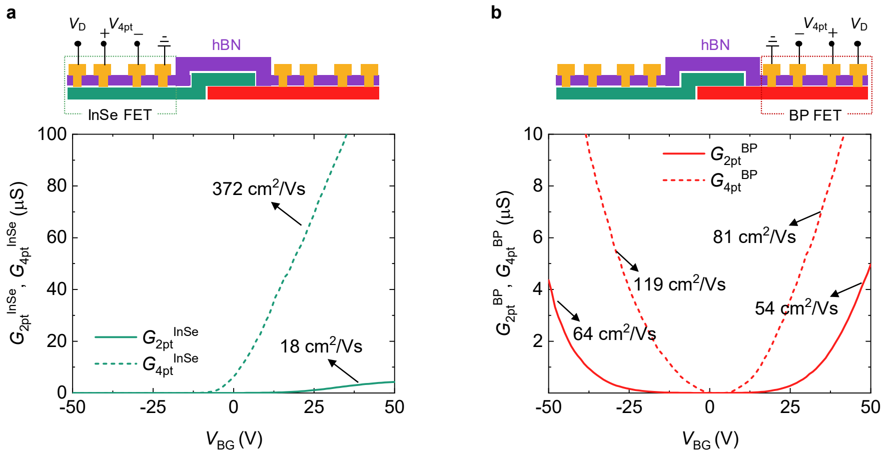

Figure S1. a) G_{2pt}^{InSe} and G_{4pt}^{InSe} measured as a function of V_{BG} at $V_D = 1$ V in a linear plot, showing the two-terminal and four-terminal field effect mobilities of the InSe. b) G_{2pt}^{BP} and G_{4pt}^{BP} in a linear plot, showing the two-terminal and four-terminal mobilities of the BP. Above schematics describe the setups for the four-terminal measurements on the InSe and BP, respectively.

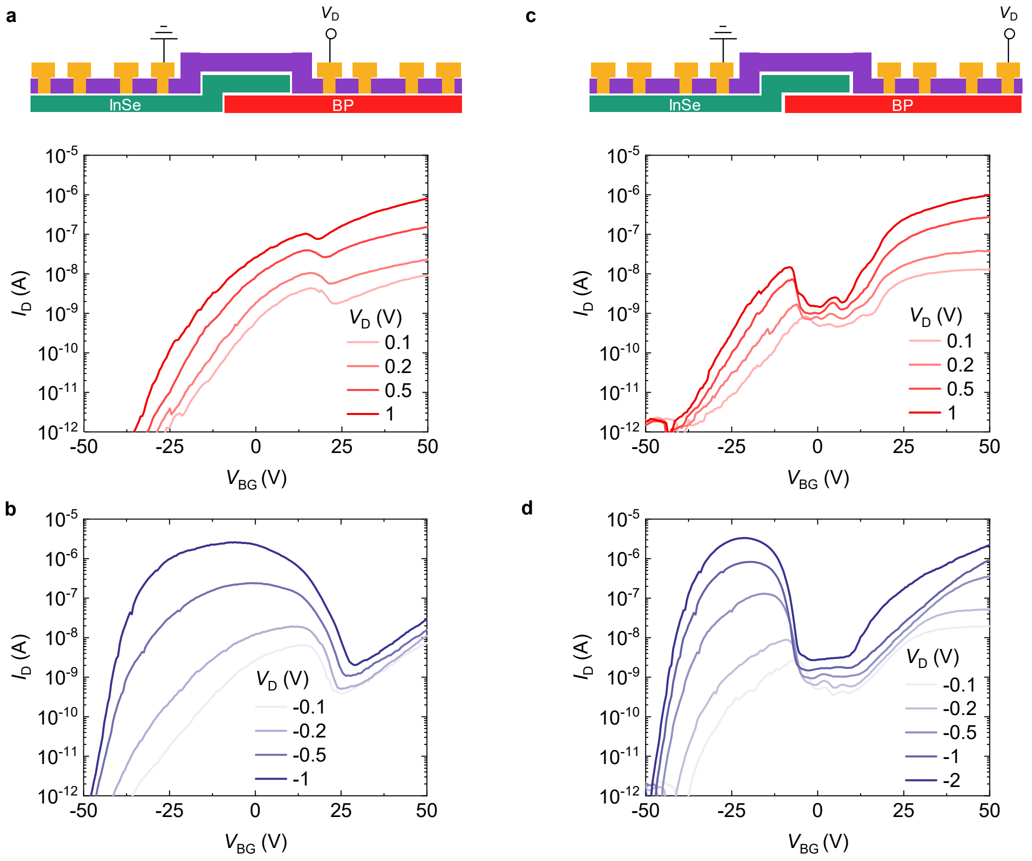

Figure S2. Transfer characteristics of additional InSe-BP-Ti device. a,b) I_D vs V_{BG} at positive V_D and negative V_D, respectively. c,d) I_D vs V_{BG} at positive V_D and negative V_D, respectively. a,b) data and c,d) data were acquired using different sets of electrodes.

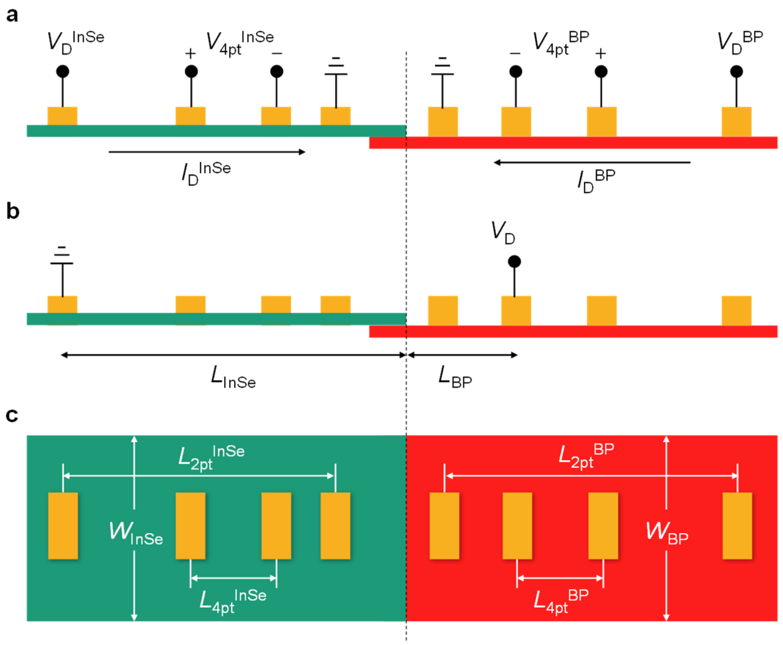

d

**InSe transistor**		
InSe two-terminal conductance	G_{2pt}^{InSe}	I_D^{InSe}/V_D^{InSe}
InSe channel conductance	G_{4pt}^{InSe}	$(I_D^{InSe}/V_{4pt}^{InSe})(L_{4pt}^{InSe}/L_{2pt}^{InSe})$
InSe contact resistance	R_C^{InSe}	$(1/G_{2pt}^{InSe} - 1/\ G_{4pt}^{InSe})/2$
InSe four-terminal resistivity	ρ_{InSe}	$(1/G_{4pt}^{InSe})(W_{InSe}/L_{2pt}^{InSe})$
**BP transistor**		
BP two-terminal conductance	G_{2pt}^{BP}	I_D^{BP}/V_D^{BP}
BP channel conductance	G_{4pt}^{BP}	$(I_D^{BP}/V_{4pt}^{BP})(L_{4pt}^{BP}/L_{2pt}^{BP})$
BP contact resistance	R_C^{BP}	$(1/G_{2pt}^{BP} - 1/\ G_{4pt}^{BP})/2$
BP four-terminal resistivity	ρ_{BP}	$(1/G_{4pt}^{BP})(W_{BP}/L_{2pt}^{BP})$
**InSe-BP-Ti device**		
InSe channel resistance	R_{InSe}	$\rho_{InSe}(L_{InSe}/W_{InSe})$
BP channel resistance	R_{BP}	$\rho_{BP}(L_{BP}/W_{BP})$
Two-terminal conductance	$G_{2pt}^{InSe\text{-}BP}$	I_D/V_D

Figure S3. a) Measurement scheme for the InSe and BP transistors. InSe and BP were measured separately, although V_D^{InSe} and V_D^{BP} are both marked in a schematic. b) Measurement scheme for the InSe-BP-Ti device. c) Schematic defining dimension parameters. d) Summary of the multiple parameters introduced here.

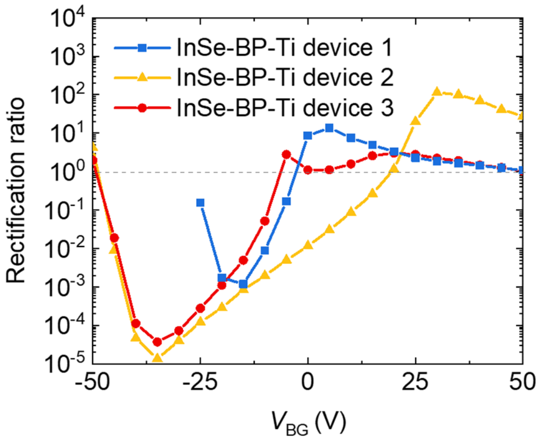

Figure S4. Rectification ratio of our multiple InSe-BP-Ti devices. Backward rectification occurs at point (*i*), while forward rectification occurs at point (*ii*). At point (*i*), current is suppressed at $V_D > 0$ (forward bias) due to the carrier depletion in the InSe near the Au/Ti contact and corresponding high R_C^{InSe}, which restrict the current injection into the channel. On the other hand, higher current can flow at $V_D < 0$ (backward bias) because electrons are injected from the BP valence band, full of electrons, via band-to-band tunneling. At point (*ii*) forward rectification occurs, similar to the conventional *p*-*n* junction.

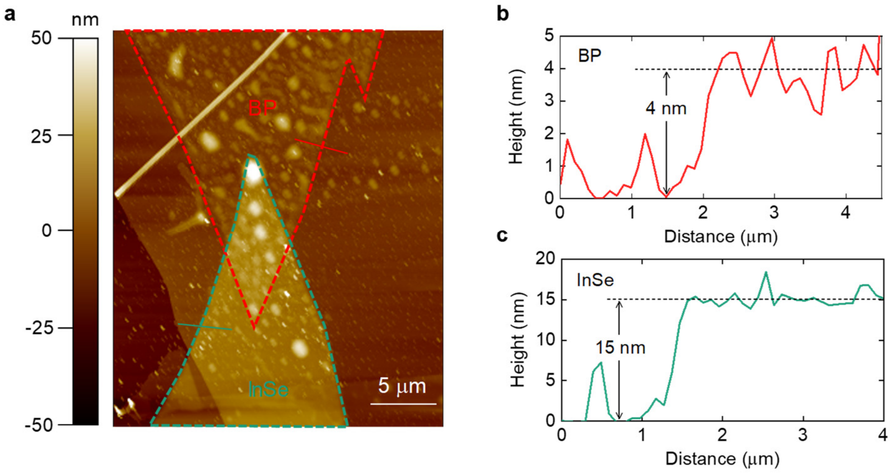

Figure S5. a) AFM image of the InSe-BP-FLG device and b,c) the line profiles showing the thicknesses of the BP and InSe flakes, respectively.

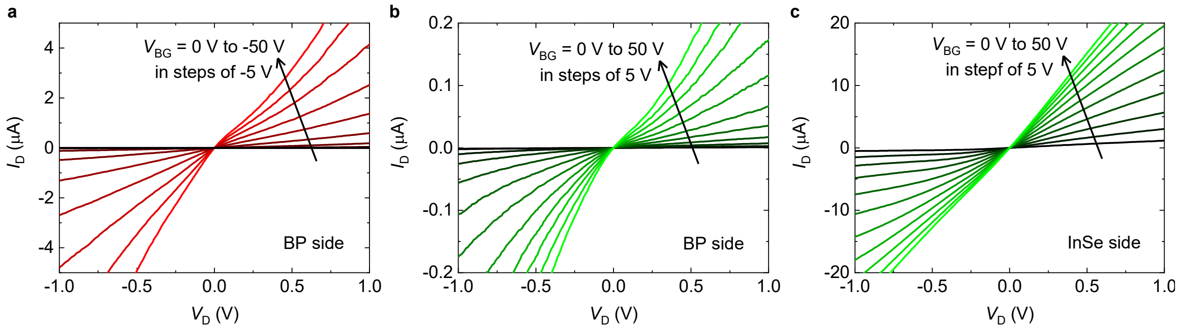

Figure S6. a,b) I_D vs V_D of the BP side and c) that of the InSe side of the InSe-BP-FLG device at different V_{BG}.

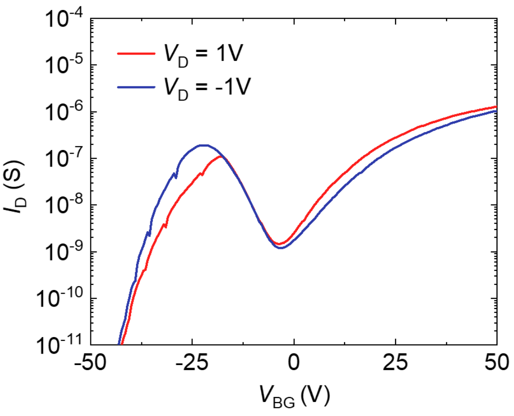

Figure S7. I_D vs V_{BG} transfer characteristics of the InSe-BP-FLG device at V_D = 1 V and -1 V.

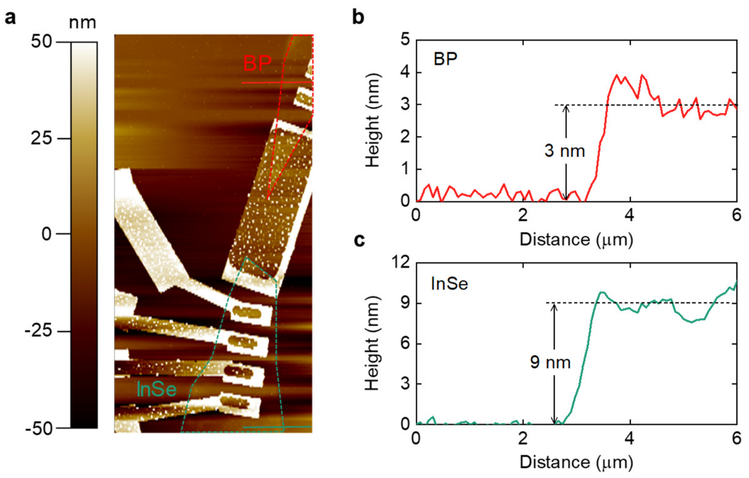

Figure S8. a) AFM image of the InSe-Ti-BP-Ti device and b,c) the line profiles showing the thicknesses of the BP and InSe flakes, respectively.

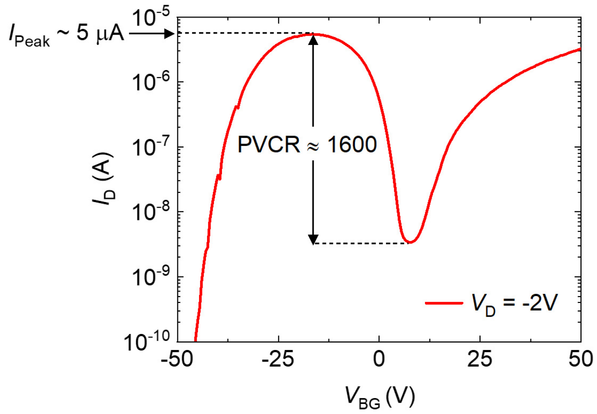

Figure S9. I_D vs V_{BG} transfer characteristic of one of our InSe-BP-Ti devices, measured at V_D = -2 V, which shows outstanding NDT performance, achieving I_{Peak} of ~5 μA and a PVCR of ~1600.
